# Supplementary material for: An exploratory study of maternal diabetes and offspring use of dental services—Northern Ireland national cohort study
Source: BDJ Open. 2023 Apr 10;9:14. doi: 10.1038/s41405-023-00140-0 (PMC10085974; doi:10.1038/s41405-023-00140-0)
Supplement: Supplementary file 1 — Supplementary Information [file 41405_2023_140_MOESM1_ESM.pdf]

Appendix 1: Additional Statistics

test xbeta1 - 1 = 0				
(1)[rawvar]xbeta1 = 1				
chi2(1) = 0.03				
Prob > chi2 = 0.8546			**n=11,116**	
	**Mean**	**Std. Err.**	**[95% Conf. Interval]**	
**Post-LD Total value of claims**	0.5787051	0.0173596	0.5446772	0.612733
**Age (month)**	111.7264	0.0325417	111.6626	111.7902
**Pre-LD prevention**	1.195124	0.0168999	1.161997	1.228251
**DEPRIVATION QUINTILE 1**	0.2471213	0.0040913	0.2391016	0.255141
**DEPRIVATION QUINTILE 2**	0.2281396	0.0039803	0.2203375	0.2359417
**DEPRIVATION QUINTILE 3**	0.2110471	0.0038704	0.2034604	0.2186339
**DEPRIVATION QUINTILE 4**	0.1876574	0.0037034	0.1803982	0.1949167
**DEPRIVATION QUINTILE 5**	0.1260345	0.003148	0.1198639	0.1322052
**Diabetes**	0.0354444	0.0017538	0.0320066	0.0388822

```
------------------------------------------------------------------------------
             |               Robust
preLDnonuser | Odds Ratio   Std. Err.      z    P>|z|     [95% Conf. Interval]
-------------+----------------------------------------------------------------
  ageinmonth |    .957383   .0003877  -107.56   0.000     .9566235    .9581431
             |
      NIMDMx |
          2  |    1.12359   .0228547     5.73   0.000     1.079677    1.169289
          3  |   1.088477   .0224112     4.12   0.000     1.045426    1.133301
          4  |   1.173174     .02494     7.51   0.000     1.125297    1.223089
          5  |    1.30998   .0308994    11.45   0.000     1.250797    1.371963
             |
       _cons |   159.2691   6.317367   127.84   0.000     147.3563    172.1449
------------------------------------------------------------------------------
Note: _cons estimates baseline odds.
```

Non-User Socioeconomic status

preLDnonuser	Odds Ratio	Std. Err.	z	P>|z|	[95% Conf.	Interval]
ageinmonth	.957383	.0003877	-107.56	0.000	.9566235	.9581431
NIMDMx						
2	1.12359	.0228547	5.73	0.000	1.079677	1.169289
3	1.088477	.0224112	4.12	0.000	1.045426	1.133301
4	1.173174	.02494	7.51	0.000	1.125297	1.223089
5	1.30998	.0308994	11.45	0.000	1.250797	1.371963
_cons	159.2691	6.317367	127.84	0.000	147.3563	172.1449

Note: _cons estimates baseline odds.

Non-user more likely to come from higher socio-economic group

Appendix 2: SDR Codes

Restoration includes the following SDR codes:
SDRcode ==1401|SDRcode ==1405| SDRcode ==1406| SDRcode ==1407| SDRcode ==1421| SDRcode ==1422| SDRcode ==1423| SDRcode ==1424| SDRcode ==1426| SDRcode ==1411| SDRcode ==4401| SDRcode ==4402| SDRcode ==1402 |SDRcode ==1403| SDRcode ==1404| SDRcode ==1408 |SDRcode ==1425| SDRcode ==1431| SDRcode ==1441 |SDRcode ==1461| SDRcode ==1471| SDRcode ==3611| SDRcode ==5001 |SDRcode ==5815| SDRcode ==5816| SDRcode ==5817 |SDRcode ==5821| SDRcode ==5822| SDRcode ==5823 |SDRcode ==5826| SDRcode ==6001| SDRcode ==6301|SDRcode == 5041

Extraction includes the following SDR codes:
SDRcode == 2101| SDRcode == 2121| SDRcode == 2201| SDRcode == 2203| SDRcode == 2221| SDRcode == 5201| SDRcode == 5206

Examination includes the following SDR codes:
SDRcode == 101| SDRcode == 111| SDRcode == 121| SDRcode == 131

Radiographs includes the following SDR codes:
SDRcode ==201| SDRcode == 202| SDRcode == 203| SDRcode == 204| SDRcode == 205| SDRcode == 301| SDRcode == 4901

Prevention includes the following SDR codes:
SDRcode ==601| SDRcode == 701| SDRcode == 1001| SDRcode == 1011| SDRcode == 4601| SDRcode == 4701

Endodontic treatment includes the following SDR codes:
SDRcode ==1501| SDRcode == 1511| SDRcode == 6311| SDRcode == 6321| SDRcode == 6331| SDRcode == 6332 |SDRcode ==6341| SDRcode == 6351 I SDRcode ==4403| SDRcode == 4404| SDRcode == 4405| SDRcode == 6003| SDRcode == 6004

Conscious Sedation includes the following SDR codes:
SDRcode ==2551| SDRcode == 2567| SDRcode == 2571| SDRcode == 2572| SDRcode == 5435| SDRcode == 5436 |SDRcode ==5451| SDRcode == 5471

Emergency includes the following SDR codes:
SDRcode ==3511| SDRcode == 3512| SDRcode == 3701| SDRcode == 5011| SDRcode == 5021| SDRcode == 5061 |SDRcode ==5601| SDRcode == 5711| SDRcode ==5712

Prescription includes the following SDR codes:
SDRcode == 3641| SDRcode == 4801| SDRcode == 2941| SDRcode == 2991
